# Aquatic bryophytes as biofilters and resource regenerators in Bioregenerative Life Support Systems: the moss on Mars project

**DOI:** 10.3389/fpls.2025.1667463

**Published:** 2025-09-23

**Authors:** Chiara Amitrano, Carmen Arena, Stefania De Pascale, Mariagabriella Pugliese, Fabrizio Barozzi, Francesco Paolo Fanizzi, Veronica De Micco, Gian Pietro di Sansebastiano

**Affiliations:** ^1^ Department of Agricultural Sciences, University of Naples Federico II, Portici (Naples), Italy; ^2^ Department of Biology, University of Naples Federico II, Naples, Italy; ^3^ Department of Physics, University of Naples Federico II, Naples, Italy; ^4^ Department of Biological and Environmental Sciences and Technologies (Di.S.Te.B.A.), University of Salento, Lecce, Italy

**Keywords:** antioxidants, aquatic bryophytes, biofiltration, bioregenerative life support systems, photosynthesis, *Leptodictyum riparium*, *Taxiphyllum barbieri*, *Vesicularia montagnei*

## Abstract

**Introduction:**

Bioregenerative Life Support Systems (BLSSs) are closed-loop systems that rely on biological processes, primarily involving plants, algae, and microbes, for sustaining long-term space missions by regenerating essential resources and recycling waste. To reduce dependency on resupply from Earth, these systems require highly efficient biological components capable of performing multiple ecological functions in constrained environments. However, research on potential BLSS components has so far focused predominantly on higher plants and algae, with aquatic bryophytes largely overlooked despite their physiological resilience, simple cultivation, and multifunctional ecological roles. This gap limits the diversification of biological components available for optimizing BLSS efficiency.

**Methods:**

Here, we investigate for the first time the potential This study investigates the potential introduction of aquatic bryophytes (mosses), specifically *Taxiphyllum barbieri*, *Leptodictyum riparium*, and *Vesicularia montagnei*, as biofilters and resource regenerators in BLSSs. Known for their adaptability, simplicity of growth, and high surface-to-volume ratio, mosses are promising candidates for controlled-environment applications. This paper characterizes mosses' performance considering gas-exchange, chlorophyll fluorescence, antioxidant activity, and biofiltration efficiency under two different controlled temperature and light conditions (24°C and 600 μmol photons m^-2^s^-1^, 22°C and 200 μmol photons m^-2^s^-1^) to determine the most suitable species for the abovementioned purposes.

**Results:**

Results indicate that *T. barbieri* exhibits the highest photosynthetic efficiency, pigment concentration, and a good biofiltering capacity, making it a promising candidate for integration into BLSSs. Notably, *L. riparium* exhibited the most effective removal of nitrogen compounds (e.g., total ammonia nitrogen) and heavy metals such as Zn, suggesting a complementary role in water purification within BLSSs.

**Discussion:**

These findings support the utilization of bryophytes in closed-loop ecological systems, with implications for both extraterrestrial and terrestrial applications. By exploring the potential of aquatic mosses, this research offers a novel and potentially advantageous biological component for enhancing the efficiency and safety of space bioreactors. These insights pave the way for future research on moss performance under prolonged stressors, including ionizing radiation, in space-like environments.

## 1 Introduction

Sustaining human life in space for extended periods presents significant challenges, particularly in providing essential resources such as oxygen, water, and food (Lasseur et al., 2010). Ongoing research focuses on developing Bioregenerative Life Support Systems (hereafter indicated as BLSS but also recently referred to as BLiSS) for future Moon and Mars missions, emphasizing the use of both transported and *in-situ* planetary resources (Russomano, 2017). Given the long duration of such missions, relying solely on Earth-based supplies for essentials like food, oxygen, and water would be impractical and prohibitively expensive (De Micco et al., 2023). To address these challenges, BLSSs aim to establish closed-loop systems that rely on biological components for resource regeneration. A key initiative in this area is the European Space Agency (ESA) MELiSSA (Micro-Ecological Life Support System Alternative) program, launched in 1989. This collaborative effort, involving ESA and several international research institutions (including the University of Naples Federico II), is focused on modeling and advancing closed-loop BLSSs critical for long-duration space missions (Mergeay et al., 1988). BLSSs consist of interconnected compartments, each housing organisms that utilize waste from other compartments as essential resources. These compartments fall into three main categories: biological producers, consumers, and waste degraders/recyclers (De Micco et al., 2023). While higher plants have traditionally been the focus of BLSSs research due to their ability to produce oxygen, remove carbon dioxide, recycle waste and water, and provide edible biomass (Kane and Shasteen, 2024), alternative biological systems, such as algae, have gained interest due to their high photosynthetic efficiency, rapid growth rates, and ability to thrive in controlled environment. Species like *Chlorella vulgaris* and *Spirulina platensis* have been investigated for their potential to produce oxygen, fix carbon dioxide, and serve as a nutritious food source for astronauts (Mussagy et al., 2024). In addition to oxygen production and food supply, algae contribute to water purification through biofiltration, removing excess nutrients and contaminants from the system, making them particularly valuable in closed-loop systems, where resource recycling is essential for long-term space missions.

However, the use of algae in BLSSs is controversial mainly due to i) biofilm formation, which can clog filtration systems, reducing light penetration, preventing gas exchange within bioreactors (Niederwieser et al., 2018; Yang et al., 2019); ii) contamination and microbial competition, which can proliferate and compete with algae for resources, potentially compromising productivity and altering system stability (Koehle et al., 2023); iii) excessive oxygen accumulation in a sealed system can lead to hyperoxia, negatively affecting other biological components, including plants and astronauts (Lasseur et al., 2010). Additionally, ensuring uniform light distribution in dense algal cultures is critical, as suboptimal lighting conditions can lead to uneven growth, with surface layers receiving excess light while deeper layers suffering from light limitation (Niederwieser et al., 2018). In this context, aquatic and semi-aquatic bryophytes offer benefits like algae, bacteria, and fungi, but without some of their drawbacks. Unlike vascular plants, bryophytes lack true roots, stems, and leaves, relying on surface absorption to obtain nutrients and water. This characteristic makes them efficient biofilters, capable of removing contaminants from water and air while contributing to oxygen production and carbon sequestration (Singh and Choudhary, 2025). Bryophytes play a crucial role in ecological succession, soil formation, and moisture retention in terrestrial ecosystems. Their ability to bind heavy metals and other pollutants has been well-documented in laboratory (Papadia et al., 2020) and in environmental remediation (Desai et al., 2025; Marat, 2025) studies, suggesting their potential for use in extraterrestrial habitats where resource recycling and waste management are critical (Marat, 2025). Furthermore, their adaptability to extreme conditions, such as low temperatures, desiccation, and high radiation levels, makes them particularly suitable for space environments.

Aquatic bryophytes have garnered attention for their role as bioindicators (Mahapatra et al., 2019; Mentese et al., 2021) and their potential in biotechnological applications, such as the production of phenols, peptides, and sugar derivatives (Mishra et al., 2014; Decker and Reski, 2020; Horn et al., 2021; Valeeva et al., 2022). Peat moss (Sphagnum spp.) is the primary industrial source of bryophyte biomass, it is extracted from pre-existing peatlands and is not a renewable resource because of its slow growth and stringent environmental conditions. Different aquatic moss species grow faster than most of other mosses, even under artificial conditions, and can be an interesting component for regenerative life support systems. However their cultivation and performance in space conditions has never been explored.

Mosses are well-suited for BLSSs because they have minimal requirements, a long life cycle, and, in some cases develop clonal lines lacking sexual reproduction, meaning they do not release spores or gametes into the water (Anglana et al., 2024). They also have potential for phytoremediation and pollution control, accumulating heavy metals and acting as natural biofilters (Gecheva and Yurukova, 2014; Papadia et al., 2020; De Matteis et al., 2021; Barukial and Hazarika, 2022). Although not used for food production, mosses offer benefits as oxygen producers, biofilters and high-quality substrates for higher plants, potentially replacing peat moss in commercial applications. However, their potential remains underexplored. Moreover, since these mosses are primarily valued for their bio-filtering capabilities or ornamental appeal, their carbon assimilation processes and the functional status of their photosystems have rarely, if ever, been investigated.

This study focuses on evaluating the potential of three aquatic bryophytes: *Taxiphyllum barbieri*, *Leptodictyum riparium*, and *Vesicularia montagnei* as bio-filters and resource regenerators in BLSSs. Among aquatic bryophytes, these species offer diverse functional traits relevant to BLSS applications. *T. barbieri* (Java moss) is valued for its adaptability to a wide range of aquatic conditions, *L. riparium* for its cosmopolitan distribution and tolerance to extreme environments, and *V. montagnei* (Christmas moss) for its dense, branched fronds that maximize surface area for biofiltration. This diversity suggests potential complementarity when multiple ecological functions are needed in closed-loop systems. By assessing their gas-exchange capacity, chlorophyll fluorescence responses, antioxidant profiles, and heavy metals and nitrogen compounds biofiltration efficiencies under two different controlled conditions, we aim to identify the most suitable species for integration into future space missions. The results of this research will contribute to understanding how non-vascular plants can enhance the sustainability of long-duration human spaceflight and may also have applications for terrestrial bioremediation efforts.

## 2 Materials and methods

### 2.1 Moss species and experimental conditions

Three aquatic bryophyte species *Taxiphyllum barbieri* (TB), *Leptodictyum riparium* (LR), and *Vesicularia montagnei* (VM) were purchased semi-axenic (after *in vitro* cultivation) from Green Greener srl (Policoro, Italy).

*T. barbieri*, commonly known as “Java moss,” originates from Southeast Asia and is one of the most widely recognized members of the *Taxiphyllum* genus. It thrives in diverse aquatic environments, including mildly brackish waters, and adapts well to a broad spectrum of light intensities and temperatures (Anglana et al., 2024). *L. riparium* is a cosmopolitan species, with a distribution that spans most of the globe except for the Pacific islands and Australia. It has been observed in extreme environments such as acid mining lakes and volcanic crater zones, tolerating pH levels as low as 1.6 (Geller et al., 2012; Anglana et al., 2024). *V. montagnei* is commonly known as “Christmas moss” for its branched fronds that resemble the shape of a Christmas tree. It is native to tropical Asia and is frequently found in humid, shaded environments such as riverbanks and forested streams. It is widely appreciated in aquascaping for its dense growth and ornamental appearance. It thrives in submerged or semi-submerged conditions under moderate light and temperature (Taeprayoon et al., 2024; Anglana et al., 2024).

For the experimental need, the three species were cultivated at the University of Salento in transparent boxes with microfilter-sterilized water (60μS/cm) in two different growth chambers under different conditions of temperature and light intensity: (i) 24°C and 600 µmol photons m^-2^s^-1^, and (ii) 22°C and 200 µmol photons m^-2^s^-1^ with a 16/8 h photoperiod. Microfilter-sterilized water was obtained from tap water to reduce mineral content and any relevant contaminants (ICP analysis in **Supplementary Table S1**), but still allowing vital processes for moss and its microbiota. The two environmental conditions were selected to represent two different environments, with the first being more demanding in terms of both temperature and light intensity, thus in terms of energy expenditure, compared to the second one. These two contrasting regimes were chosen to reflect possible realistic operational ranges for controlled-environment BLSS modules, ensuring that the resulting data are directly relevant to system design and management. While they do not allow the determination of precise physiological optima, they provide an effective first screening for identifying species-specific functional differences under conditions representative of potential BLSS scenarios. All physiological, and biochemical analyses were conducted on all three species (*Taxiphyllum barbieri*, *Leptodictyum riparium*, and *Vesicularia montagnei*). Nitrogen biofiltration assays were performed after 12 days only on *T. barbieri* and *L. riparium*, as *V. montagnei* exhibited insufficient biomass accumulation and growth stability under the tested conditions, which reduced the competitive applicability of this species in biofiltration performance assays. In both experimental situations the mosses were grown in non-sterile conditions but axenic starting material.

### 2.2 Photosynthetic performance

Gas-exchanges were performed on five replicates per species by a portable gas-exchange system (LCpro+, ADC BioScientific, UK), using the ADC versatile chamber. The ADC Versatile Chamber is a chamber incorporating an enclosed volume used for the measurement of whole small plant/algae/mosses/soil gas-exchanges. The Versatile Chamber consists of an acrylic pot containing an air stirrer fan and pressure equalization vent situated on the sides of the pot. The upper surface is entirely clear and transparent to maximize the light transmission to the sample within the chamber. Before measurements, specimens were gently blotted on moist filter paper to remove excess surface water, minimizing the formation of a water film that could impede gas exchange while avoiding desiccation. Measurements were performed immediately after transferring the specimens into Petri dishes placed inside the chamber, with a continuous flow of ambient air (CO_2_ ≈ 400 ppm) to ensure stable CO_2_ availability. Measurements were carried out at 25 ± 2°C leaf temperature and 500 µmol m^−2^s^−1^ photosynthetic photon flux density (PPFD). The relative humidity in the leaf chamber was set at 50–60%. From gas-exchanges, net-assimilation and transpiration rates were recorded after reaching a steady-state, after 10–15 minutes per sample, and calculated by the equations of Von Caemmerer and Farquhar (1981) within the software operating with the gas-exchange system.

### 2.3 Photosynthetic pigment content

The determination of the photosynthetic pigments content, namely total chlorophylls (a + b) and carotenoids (x + c), was performed according to Lichtenthaler (1987) on five replicates, collected from the different treatments per species from the two experimental conditions, considering one individual as one replicate. Frozen samples of the known weight (0.040 g) were homogenized in ice-cold 100% acetone using a mortar and pestle. The extracts were centrifuged at 5000 rpm for 5 min in a Labofuge GL (Heraeus Sepatech, Hanau, Germany). The sample absorbance was measured by a spectrophotometer (UV-VIS Cary 100; Agilent Technologies) at wavelengths of 662 nm, 630 nm, and 470nm for chlorophyll a, chlorophyll b and total carotenoids, respectively. Pigment concentration was expressed as µg g^-1^ of dried weight (µg g^-1^ DW).

### 2.4 DPPH scavenging assay and polyphenols extraction

Polyphenol content and DPPH assay were evaluated on five replicates, collected from the different treatments per species from the two experimental conditions, considering one individual as one replicate.

The DPPH (2,2-diphenyl-1-picrylhydrazyl) assay was used to evaluate the radical scavenging activity following the procedure described in Pica et al. (2024). Briefly, 0.067 mL of methanolic extracts were added to 2mL of 6 x 10–^5^ M DPPH in methanol solution and heated at 37°C for 20 minutes in a dry bath (Benchmark scientific, My block™ Mini Dry Bath). After, absorbance was measured at a wavelength of 515 nm using a UV-VIS spectrophotometer (UV-VIS Cary 100; Agilent Technologies). Antioxidant capacity was assessed using Trolox as positive control and expressed as percentage of radical inhibition, as shown in Equation 1:


(1) %inhibition=(Acontrol–Asample/Acontrol)×100

where Acontrol is the blank absorbance on the DPPH methanolic solution, and Asample is the sample absorbance (Rauf et al., 2025).

The polyphenol content was evaluated through the Folin- Ciocalteu method following the procedure reported in Donadio et al. (2025). Briefly, fresh samples (0.20 g) were powdered in liquid nitrogen, treated with 80% methanol at 4°C and centrifuged at 11,000 rpm for 5 min. Extracts were mixed with 10% Folin Ciocâlteu reagent (1:1, v:v) and 700 mM Na2CO3 solution (1:5, v:v), and incubated for 2 h in the darkness at room temperature. The absorbance was measured at 765 nm with a spectrophotometer (UV-VIS Cary 100; Agilent Technologies). The total polyphenol content was expressed as mg of Gallic Acid Equivalents g^-1^ DW (mg GAE g^-1^ DW) using a gallic acid standard curve.

### 2.5 Metal filtration assay

Biomasses of 500 mg of live moss from the 2 species with better physiological performance, TB and LR were incubated in 50 mL Falcon tubes containing 25 mL of 60μS/cm water contaminated with Cu^2+^ (CuSO4 4 μM), Pb^2+^ (Pb(NO3)2 100 μM) and Zn^2+^ (ZnSO4 40 μM), determining the concentrations showed in **Supplementary Table S1**. At the beginning of each experiment replica (time 0), after 24, 48 and 72 hours, 2 mL of liquid were collected from each Falcon. 1.0 ml of each liquid sample collected, was acidified and diluted to a final volume of 10 mL with HNO3 (2% v/v). All samples were analyzed using ICP/AES (iCAP 6000, Thermo Scientific, Waltham, MA, USA). The spectrometer was previously calibrated for quantitative analysis with five different concentration (0.001, 0.01, 0.1, 0.5, and 1.0 mg/L.) of standard solutions (Ultra Scientific Analytical Solution, Bologna, Italy) containing known concentrations of the elements: The calibration lines showed correlation coefficients (r) greater than 0.99 for all the measured elements. The results were expressed as the average of three different measurements, showing the decrease of Cu, Pb, and Zn concentrations as percentage over starting concentration. Lacking the analysis of uptake in the biomass, we refer to this reduction effect as bioremediation. All experiments were repeated independently 3 times per replicate. The percentage value reported was calculated based on the sample value at time 0.

### 2.6 Total ammonia nitrogen filtration assay

Biomasses of 250 mg of live moss from the 2 species with better physiological performance, TB and LR, were incubated in 500 ml Magenta boxes containing 250 ml of ammonia nitrogen solution prepared to simulate heavily urine-polluted (about 10%) wastewater, consisting of 100 mM NH_4_Cl and 50 mM urea dissolved in 60 μS/cm water 100 mM NH_4_Cl and 50 mM of urea dissolved in 60μS/cm water. Continuous aeration (0.5 L min^-1^) using air stones was applied for each Magenta box. At the beginning of each experiment replica (time 0), after 48, 120 and 280 hours, 2 mL of liquid were collected from each Magenta box: Total ammonia nitrogen (TAN) was measured with the Hydrocheck Spectratest kit (Sheffield, UK) based on a colorimetric method revealing ammonia in solution. The concentration of TAN was measured spectrophotometrically using a Shimadzu 2600 UV-Visible spectrophotometer and decrease was expressed as percentage of starting concentration. Lacking the analysis of uptake in the biomass, we refer to this reduction effect as bioremediation.

### 2.7 Statistical analysis

Statistical analyses were carried out using R (version 4.4.3), employing the *stats* and *agricolae* packages. To assess species-specific differences in physiological and biochemical traits, one-way ANOVAs were performed independently within each environmental condition. Tukey’s HSD *post hoc* tests were used to detect pairwise differences among species. Additionally, two-way ANOVAs were conducted as supplementary analyses to explore the main and interactive effects of species and environment (**Supplementary Table S2**). Also, for biofiltration-related traits, a three-way ANOVA was conducted to evaluate the individual and combined effects of species, environmental condition, and time of exposure (**Supplementary Table S3**). *Post hoc* comparisons were carried out using Tukey’s test at a significance threshold of p ≤ 0.05. Radar plots were generated in R using the *fmsb* and *ggplot2* packages. To enable comparison across traits with different units and scales, all physiological parameters were normalized using a min–max transformation, a standard procedure in radar plots:


x′=x–xmin/max​−xmin​x

This rescaled all values between 0 (minimum observed value) and 1 (maximum observed value) for each trait, allowing a direct visual assessment of the relative performance of each species under the two environmental conditions without bias from unit or scale differences.

## 3 Results

### 3.1 Photosynthetic performance

**Figure 1:**
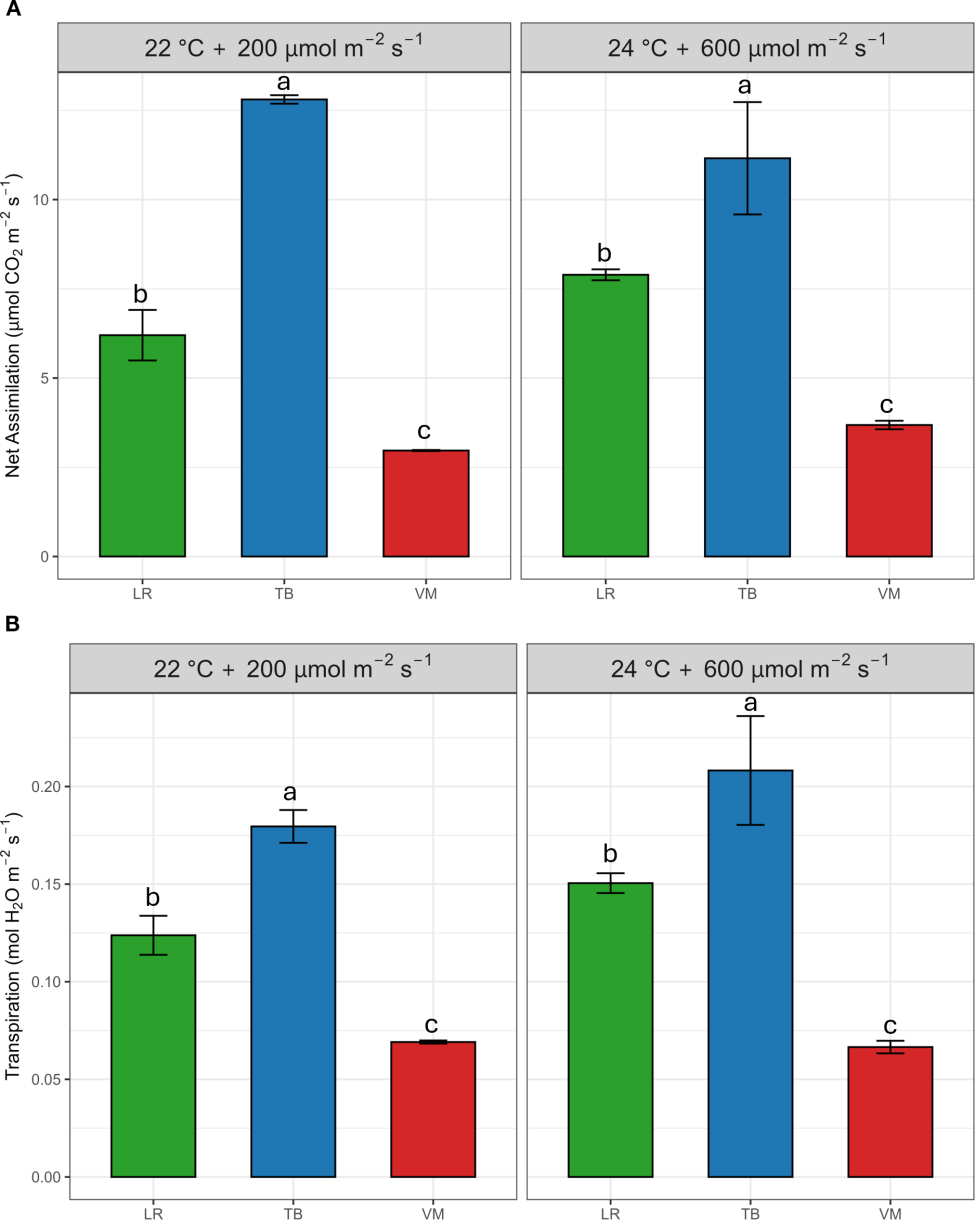
Net photosynthetic rate **(A)** and transpiration rate **(B)** of the three moss species (*Leptodictyum riparium* LR, *Taxiphyllum barbieri* TB, and *Vesicularia montagnei* VM) under two environmental conditions: 22°C + 200 µmol m^-2^ s^-1^ and 24°C + 600 µmol m^-2^ s^-1^. Bars represent mean ± standard error. Different letters indicate statistically significant differences among species within each environmental condition (Tukey’s HSD test, p< 0.05).

Net-assimilation and transpiration rates differed significantly among species under both environmental conditions (**Figures 1A, B**). *T. barbieri* (TB) exhibited the highest net assimilation rate in both environments, significantly outperforming *L. riparium* (LR) and *V. montagnei* (VM). A similar trend was observed for transpiration. TB displayed the highest transpiration rates across both environments, whereas LR and VM had significantly lower values. These differences were supported by the two-way ANOVA (**Supplementary Table S2**), which revealed highly significant species effects (p< 0.001), for both net-assimilation and transpiration rates, while environmental effects and species × environment interactions were not significant.

### 3.2 Photosynthetic pigment content

**Figure 2:**
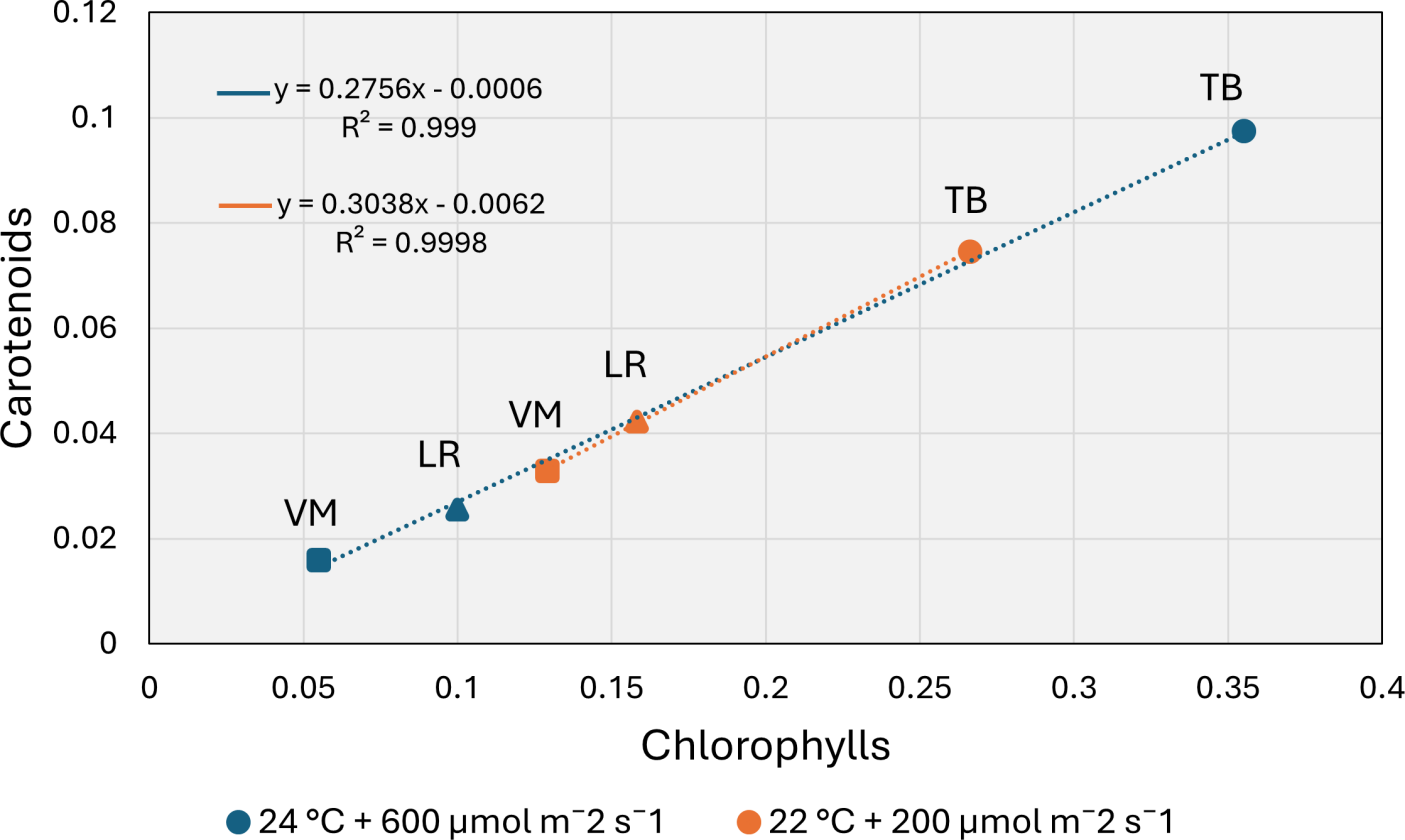
Relationship between total chlorophylls and carotenoids in the three moss species: *Taxiphyllum barbieri* (TB, circles), *Leptodictyum riparium* (LR, triangles), and *Vesicularia montagnei* (VM, squares), under two environmental conditions: 22°C + 200 µmol m^-2^ s^-1^ (orange) and 24°C + 600 µmol m^-2^ s^-1^ (blue). Each point represents species mean values for pigment content. Linear regression lines and equations are shown for each condition. The strong positive correlation (R² > 0.99) highlights a coordinated accumulation of chlorophylls and carotenoids across species and conditions.

Under both environmental conditions, *Taxiphyllum barbieri* (TB) showed the highest concentrations of photosynthetic pigments compared to the other two moss species (**Table 1**). At 22°C and 200 µmol m^-2^ s^-1^, TB exhibited significantly higher contents of chlorophyll a (0.17 ± 0.003 mg g^-1^ FW), chlorophyll b (0.09 ± 0.002 mg g^-1^ FW), total chlorophylls (0.26 ± 0.004 mg g^-1^ FW), and carotenoids (0.07 ± 0.001 mg g^-1^ FW) than *Leptodictyum riparium* (LR) and *Vesicularia montagnei* (VM). In particular, VM displayed the lowest carotenoid content, significantly different from both TB and LR (p < 0.01). At 24°C and 600 µmol m^-2^ s^-1^, pigment content increased in TB but decreased further in LR and VM. TB again showed the highest values for chlorophyll a (0.23 ± 0.001 mg g^-1^ FW), chlorophyll b (0.13 ± 0.001 mg g^-1^ FW), total chlorophylls (0.35 ± 0.014 mg g^-1^ FW), and carotenoids (0.09 ± 0.003 mg g^-1^ FW). Conversely, VM recorded the lowest pigment concentrations across all parameters, with total chlorophylls dropping to 0.05 ± 0.002 mg g^-1^ FW and carotenoids to 0.02 ± 0.001 mg g^-1^ FW (p < 0.001). Two-way ANOVA (**Supplementary Table S2**) confirmed a highly significant effect of species for all pigments (p < 0.001), and revealed significant species × environment interactions for chlorophyll a, chlorophyll b, total chlorophylls, and carotenoids (p < 0.001). The environmental factor alone had a weaker or non-significant influence, except for total chlorophylls (p = 0.041), suggesting that pigment accumulation is primarily species-dependent, but modulated by environmental conditions in an interactive manner. **Figure 2** displays the relationship between total chlorophyll content and carotenoid concentration across all species and environmental conditions. A strong positive correlation was observed for both environments (R² = 0.99), indicating that carotenoid accumulation is closely associated with chlorophyll content. When dissecting the distribution by condition, it becomes evident that LR and VM accumulated higher pigment levels at 22°C, showing a marked decrease in total chlorophylls and carotenoids under 24°C and 600 µmol m^-2^ s^-1^. In contrast, TB maintained or even enhanced its pigment levels under higher light and temperature, occupying the upper range of the regression curve regardless of condition.

**Table 1:** Photosynthetic pigment concentration in *Leptodictyum riparium* LR, *Taxiphyllum barbieri* TB*, Vesicularia montagnei* VM growing in two different environments (24°C + 600 µmol m^-2^ s^-1^ and 22°C + 200 µmol m^-2^ s^-1^).

Treatments	Chlorophyll a	Chlorophyll b	Total Chlorophylls	Carotenoids	
LR_22°C + 200 µmol m^^-^2^ s^^-^1^	0.11 ± 0.012 *c*	0.05 ± 0.003 *b*	0.16 ± 0.001 *b*	0.04 ± 0.001 *b*
TB_22°C + 200 µmol m^^-^2^ s^^-^1^	0.17 ± 0.003 *a*	0.09 ± 0.002 *a*	0.26 ± 0.004 *a*	0.07 ± 0.001 *a*
VM_22°C + 200 µmol m^^-^2^ s^^-^1^	0.08 ± 0.001*c*	0.05 ± 0.001 *b*	0.13 ± 0.007 *b*	0.03 ± 0.001 *c*
LR_24°C + 600 µmol m^^-^2^ s^^-^1^	0.06 ± 0.006 *b*	0.03 ± 0.004 *b*	0.10 ± 0.005 *b*	0.02 ± 0.001 *b*
TB_24°C + 600 µmol m^^-^2^ s^^-^1^	0.23 ± 0.001 *a*	0.13 ± 0.001 *a*	0.35 ± 0.014 *a*	0.09 ± 0.003 *a*
VM_24°C + 600 µmol m^^-^2^ s^^-^1^	0.03 ± 0.001 *c*	0.02 ± 0.001 *c*	0.05 ± 0.002 *c*	0.02 ± 0.001 *b*
Signfificance				
Species	***	***	***	***
Environment	***	*ns*	*	*ns*
Species x Environment	**	*ns*	**	*ns*

Means ± standard errors are shown with different letters indicating significant differences of the interaction, according to Tukey *post hoc* test. Results of two-way ANOVA testing the effects of species, environmental condition, and their interaction is also reported. Significance codes: ns, non significant, p ≤ 0.001 (***), p ≤ 0.01 (**), p ≤ 0.05 (*).

### 3.3 DPPH assay and polyphenols

**Figure 3:**
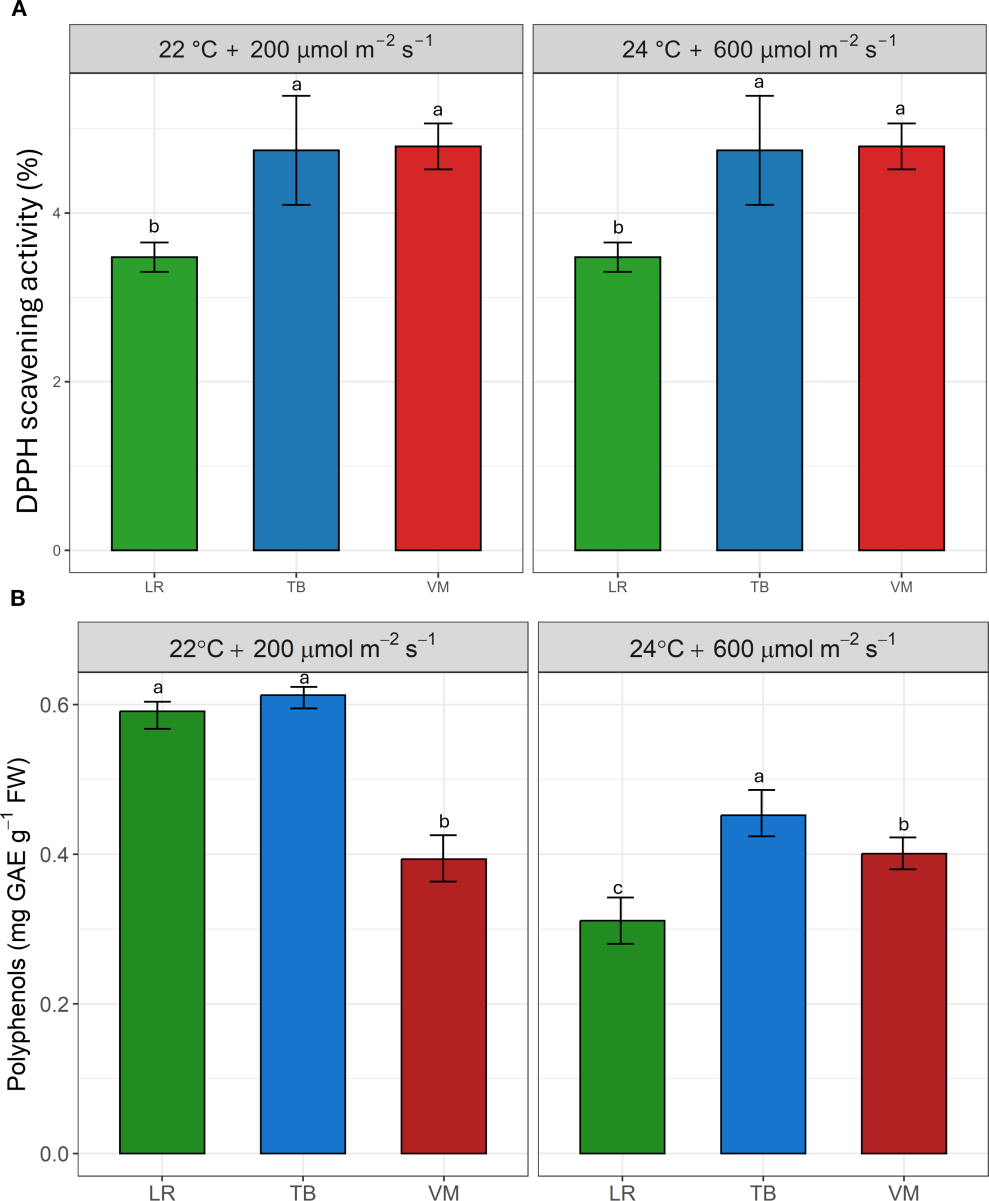
Antioxidant capacity (expressed as DPPH scavenging activity, %, **(A)** and total polyphenol content (expressed as mg gallic acid equivalents [GAE] g^-1^ fresh weight, **(B)** in the three moss species (*Leptodictyum riparium* LR, *Taxiphyllum barbieri* TB*, Vesicularia montagnei* VM) grown under two environmental conditions: 22°C + 200 µmol m^-2^ s^-1^ and 24°C + 600 µmol m^-2^ s^-1^. Bars represent mean ± standard errors. Different letters indicate statistically significant differences among species within each condition (Tukey’s HSD test, p< 0.05).

**Figure 3** shows the antioxidant capacity, expressed as DPPH radical scavenging activity (**Figure 3A**), and the total polyphenol content (**Figure 3B**) in the three moss species under two environmental conditions. TB and VM exhibited the highest antioxidant activity in both environments, with no statistically significant differences between them, compared to LR. The pattern for polyphenol content was slightly different: TB consistently showed the highest values across both environments. At 22°C and 200 µmol m^-2^ s^-1^, no significant difference was observed between TB and LR, whereas VM showed a decrease in polyphenol levels. At 24°C and 600 µmol m^-2^ s^-1^, LR had significantly lower polyphenol content compared to VM which in turn showed significantly lower values than TB.

### 3.4 Characterization of moss physiological activities

**Figure 4:**
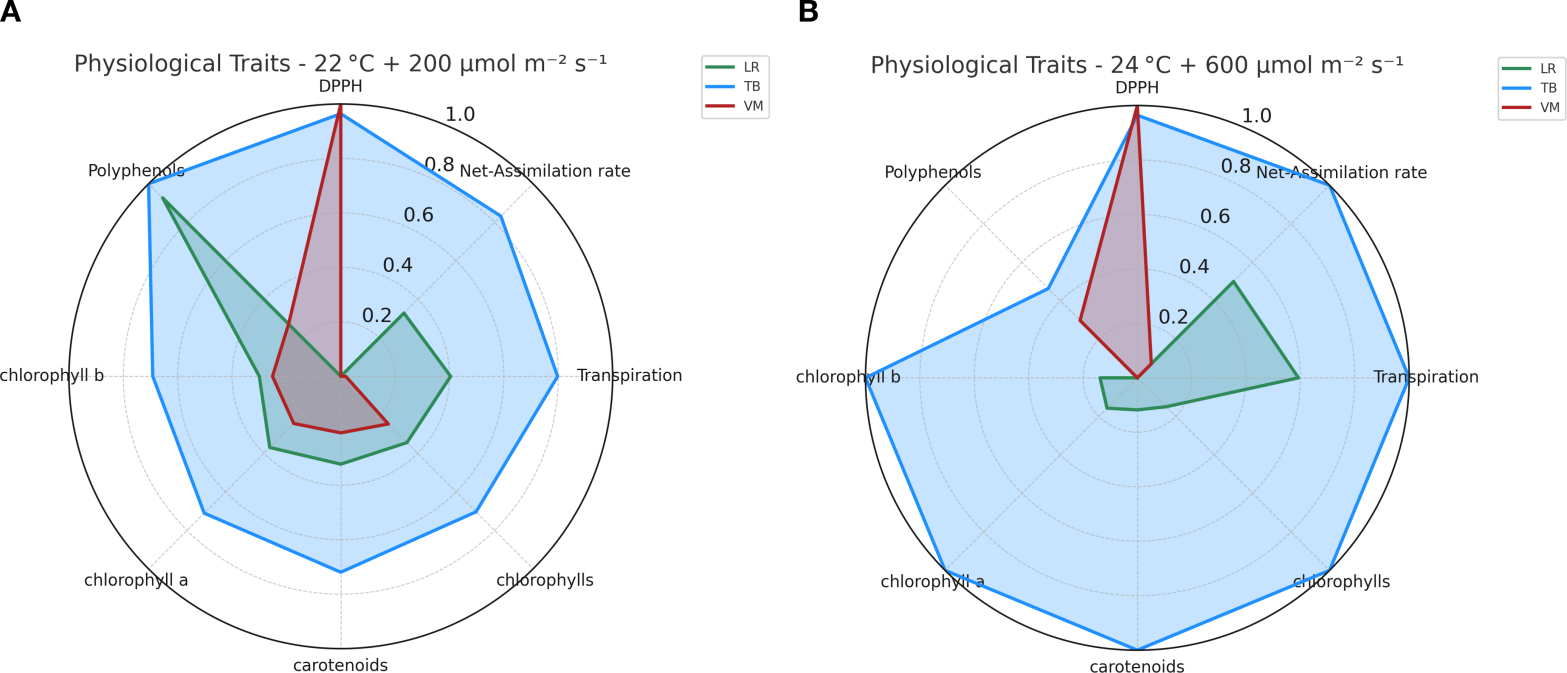
Radar plots of normalized physiological and biochemical traits in the three moss species (*Leptodictyum riparium* LR, *Taxiphyllum barbieri* TB, *Vesicularia montagnei* VM) under two environmental conditions. **(A)** Trait profiles under 22°C + 200 µmol m^-2^ s^-1^. **(B)** Trait profiles under 24°C + 600 µmol m^-2^ s^-1^. Traits include net photosynthetic rate, transpiration rate, total chlorophylls, chlorophyll a, chlorophyll b, carotenoids, total polyphenols, and DPPH scavenging activity. Each variable was scaled between 0 and 1 based on the highest recorded value across all samples.

The physiological profiles of the three moss species (LR, TB, VM) were compared using radar plots that integrated multiple physiological traits and visually highlight the separation among species based on their overall physiological trait expression. Under moderate light and temperature of 22°C + 200 µmol m^-2^ s^-1^ (**Figure 4A**), TB exhibited the most balanced and efficient physiological profile, with the highest values in net photosynthesis, pigment concentration, and DPPH activity. LR showed good chlorophyll content, but lower photosynthetic performance and antioxidant capacity compared to TB. VM had the weakest profile, with uniformly low values across all traits but DPPH. Under higher light and temperature 24°C + 600 µmol m^-2^ s^-1^ (**Figure 4B**), TB further enhanced its performance, especially in net-assimilation and pigment accumulation, maintaining a dominant physiological profile. LR showed improvements in photosynthesis and carotenoids compared to the 22°C condition, but still lagged behind TB. VM remained the least responsive, with only increases in DPPH. Both radar charts (**Figures 4A, B**) highlighted TB’s consistently superior performance with high values across most parameters in both environmental conditions. LR showed intermediate values across most physiological traits, with noticeable variation between the two environmental conditions. Specifically, increases at 22°C + 200 µmol m^-2^ s^-1^ were found, except for net assimilation rate and transpiration. VM consistently displayed the lowest values for all traits, with minimal differences between the two environmental conditions, indicating a relatively stable physiological profile. Notably, polyphenol content followed a similar pattern to antioxidant activity, with TB and LR showing higher levels under moderate conditions, while VM exhibited a strong decrease at 22°C + 200 µmol m^-2^ s^-1^ and only partial recovery under 24°C + 600 µmol m^-2^ s^-1^. This trend further supports the distinct antioxidant strategies among the species and emphasizes TB’s superior capacity to activate both enzymatic and non-enzymatic defense mechanisms.

### 3.5 Bio-filtering capacity

**Figure 5:**
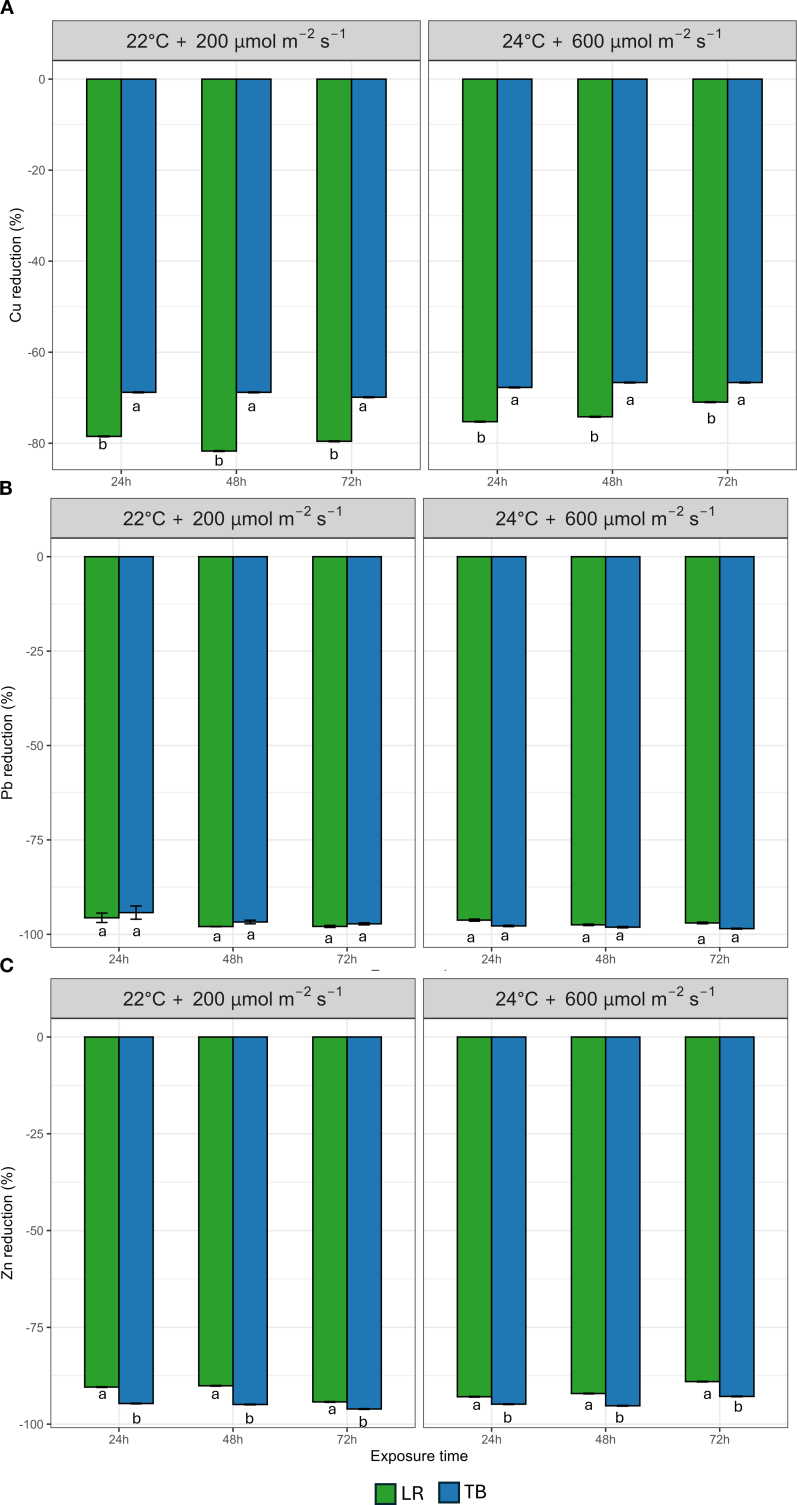
Percentage decrease of heavy metals **(A)** Copper (Cu), **(B)** Lead (Pb), and **(C)** Zinc (Zn) after 24 h, 48 h, and 72 h in two moss species (*Leptodictyum riparium* LR and *Taxiphyllum barbieri* TB) grown under two environmental conditions: 22°C + 200 µmol m^-2^ s^-1^ and 24°C + 600 µmol m^-2^ s^-1^. Bars represent mean ± standard errors. Different letters indicate statistically significant differences among species within each condition (Tukey’s HSD test, p< 0.05).

Willing to explore mosses bio-filtration capacities, we did not distinguish between uptake or adsorption of pollutants but measured solely the decrease of pollutants in the contaminated water, referring to it as a bioremediation effect. The HMs and ammoniacal nitrogen contaminations were tested separately. **Figures 5A–C** shows data on the percentage decrease of Cu (**Figure 5A**), Pb (**Figure 5B**), and Zn (**Figure 5C**), across three exposure times (24, 48, and 72h) under the two environmental conditions explored (22°C + 200 µmol m^-2^ s^-1^ and 24°C + 600 µmol m^-2^ s^-1^). Cu decrease showed marked species-specific differences. LR consistently exhibited significantly higher Cu decrease % than TB at all time points and under both environmental conditions. LR achieved overall a Cu decrease exceeding 60%, while TB remained below 50%. Differently, Pb decrease was uniformly high across treatments, approaching or exceeding 95% in both species. Although both mosses showed comparable filtration capacity, statistical analysis revealed a significant effect of environmental condition and species × environment interaction, suggesting subtle differences in how each species responds to environmental variation in Pb removal efficiency. Zn decrease followed a similar pattern to Cu decrease %, with species-specific differences. Overall, TB showed a significantly higher decrease % under both conditions.

**Figure 6:**
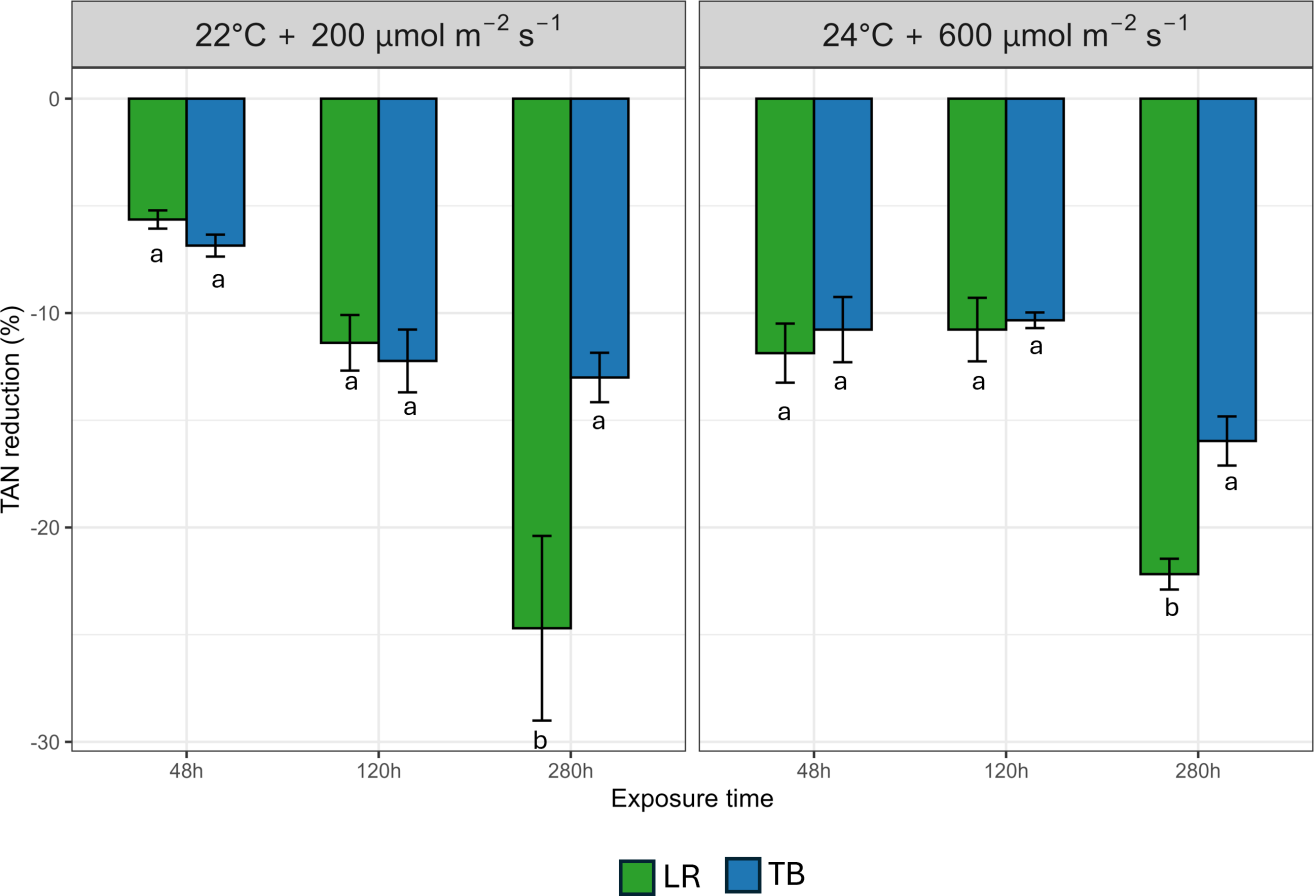
Percentage decrease of Total Ammoniacal Nitrogen (TAN) after 48 h, 120 h, and 280 h in two moss species (*Leptodictyum riparium* LR and *Taxiphyllum barbieri* TB) grown under two environmental conditions: 22°C + 200 µmol m^-2^ s^-1^ and 24°C + 600 µmol m^-2^ s^-1^. Bars represent mean ± standard errors. Different letters indicate statistically significant differences among species within each condition (Tukey’s HSD test, p< 0.05).

In **Figure 6** data about Total Ammoniacal Nitrogen (TAN) after 48 h, 120 h, and 280 h are presented. At 48 h and 120 h, both LR and TB exhibited moderate TAN decrease (between −5% and −12%), with no statistically significant differences between species under the two different environmental conditions. However, at 280 h, LR displayed a pronounced increase in TAN decrease (−25%), significantly outperforming TB (−10%) under both environments. This species-specific difference became evident only after prolonged exposure, as confirmed by the significant species × time interaction.

## 4 Discussion

Bioregenerative Life Support Systems (BLSSs) require biological components capable of performing multiple ecological functions under controlled and often resource-limited conditions. This study provides the first integrated assessment of aquatic bryophytes as potential components of BLSSs, focusing on their physiological efficiency, antioxidant activity, and biofiltration performance under controlled environmental conditions. Our comparative analysis of *T. barbieri*, *L. riparium*, and *V. montagnei* revealed distinct species-specific strategies that highlight their differential suitability for BLSSs integration. These findings highlight the potential of aquatic mosses as versatile components for space-based regenerative systems, offering both physiological resilience and functional filtering performance.

*T. barbieri* emerged as the most physiologically robust species, exhibiting the highest rates of net-photosynthesis and pigment accumulation across both environments. Its ability to maintain elevated levels of chlorophyll a, b, and carotenoids under high light and temperature suggests a well-developed photoprotective strategy conferring the photosynthetic apparatus with the capacity for rapid acclimation to variable light environments. This property is especially valuable in space-based systems, where light and thermal regimes may fluctuate due to technical constraints or mission design, and where environmental conditions can be highly controlled but also energetically expensive to maintain (Nguyen et al., 2023).

Currently, it is difficult to draw comparisons with recent literature, which mostly focuses on the physiological responses of aquatic mosses following exposure to pollutants, rather than on direct measurements of photosynthetic parameters under controlled conditions (De Agostini, 2022). However, the distinct species-specific patterns observed in pigment content and net assimilation are consistent with previous reports on aquatic moss plasticity where different species can modulate their light use efficiency differently, depending on environmental conditions, such as water depth, turbidity, flow, nutrients, and temperature (Duckett et al., 2004; Glime, 2013). In particular, in our study *T. barbieri*’s enhanced performance under high light and temperature conditions may reflect a more sun-adapted photophysiology, unlike *L. riparium* and especially *V. montagnei*, which exhibited marked declines, aligning with observations of shade-tolerant behavior in submerged bryophytes (Sand-Jensen and Madsen, 1991).

Bryophytes have traditionally been described as shade plants, but rather than being strictly shade-adapted, their light-saturation levels in sun are lower than those for vascular plants and generally exhibit lower chlorophyll a:b ratios compared to aquatic tracheophytes, features that reflect their adaptation to low-light environments rather than an obligate shade-plant strategy (Proctor, 2000; Marschall and Proctor, 2004). In line with previous observations, *L. riparium* and *V. montagnei* displayed features consistent with a shade-tolerant strategy, including lower pigment content and a relatively modest response to increased irradiance. In contrast, *T. barbieri* markedly increased both chlorophyll a and b concentrations under elevated irradiance and temperature, indicating a greater photosynthetic plasticity and potential for acclimation to a broader range of light environments. This apparent ecological specialization limits their potential under high-light BLSS conditions, unless systems are tailored to lower irradiance regimes. These findings emphasize the functional diversity within aquatic mosses and their differential potential for incorporation into BLSSs. In a recent study comparing the performance of *T. barbieri* and *V. montagnei* under heavy metal stress (Jose, 2022), *T. barbieri* exhibited a higher initial chlorophyll content and a relatively more gradual pigment degradation, suggesting more efficient physiological machinery, potentially linked to more efficient detoxification mechanisms or chloroplast protection strategies.

**Figure 2** highlights a strong positive correlation between total chlorophylls and carotenoids across all species and environments, suggesting coordinated regulation of light-harvesting and photoprotective pigments. This coordination is particularly evident in *T. barbieri*, which maintains high pigment concentrations even under elevated light and temperature, indicating a balanced investment in both photosynthetic and protective components. In contrast, *L. riparium* and *V. montagnei* cluster in the lower range of the plot, consistent with a shade-adapted phenotype with lower overall pigment levels. Interestingly, both species accumulated more pigments under the low light/low temperature environmental conditions, suggesting a potential sensitivity to higher temperature or light intensity. This pattern aligns with previous findings that aquatic bryophytes typically possess lower chlorophylls and reduced carotenoid investment compared to tracheophytes, reflecting adaptation to low-light environments (Marschall and Proctor, 2004).

In terms of antioxidant response, while *V. montagnei* presented antioxidant levels comparable to *T. barbieri*, this similarity did not translate into higher physiological performance or filtration efficiency. This finding is consistent with observation by Smolińska-Kondla et al. (2022), who reported that elevated phenolic content in bryophytes does not necessarily correlate with functional acclimation, but may reflect a generalized oxidative stress response, indicating an investment of energy into plant defense system. In fact, the elevated antioxidant activity observed in *V. montagnei* may represent a compensatory mechanism related to its low pigment content and limited photosynthetic capacity. Similar conclusions were drawn by Ćosić et al. (2023), who found species-specific patterns in the accumulation of phenolic compounds under salinity stress, emphasizing the decoupling between secondary metabolites and primary metabolism. This suggests that *V. montagnei* may rely more heavily on constitutive or stress-induced antioxidant defenses, such as phenolics, rather than photoprotection via carotenoids or chlorophyll-mediated energy dissipation, a strategy commonly seen in slow-growing or habitat-restricted bryophyte species. As an ornamental species, *V. montagnei* has been primarily selected for its aesthetic traits, such as frond architecture and growth form, rather than for functional physiological efficiency. This may partly explain its limited performance under the environmental conditions tested. These observations are coherent with the observation of a much slower growth of *V. montagnei* compared to the other two species (Anglana et al., 2024). While any apical explants of TB and LR increase length from 50% to 200% in 1 month, VM shows a very limited growth in the same time interval (not shown).

In parallel with antioxidant activity, polyphenol content also exhibited marked interspecific and environmental variation. *T. barbieri* maintained the highest levels of polyphenols across both environmental conditions, while *V. montagnei* showed a significant decrease under moderate light and temperature (22°C + 200 µmol m^-2^ s^-1^), with only partial recovery at higher irradiance and temperature. *L. riparium* accumulated high polyphenol levels under 22°C but exhibited a sharp decline under 24°C, indicating a limited capacity to maintain secondary metabolite production under thermal or light stress. Our results agree with Kumar et al. (2023), who reported that phenolic biosynthesis in plants is highly sensitive to environmental fluctuations, and can be either enhanced or suppressed depending on the stress type and severity. In *T. barbieri*, the co-occurrence of high polyphenol content, DPPH activity, and physiological performance supports the view that its antioxidant machinery is not merely compensatory but part of a robust acclimation strategy. This harmonized response may reflect a more efficient allocation of metabolic resources toward both photoprotection and stress mitigation, enhancing its potential for inclusion in BLSSs where oxidative stress (e.g., radiation, high light) is a recurrent challenge.

Interestingly, the species’ biofiltration performance revealed a functional divergence not entirely aligned with photosynthetic capacity. While *T. barbieri* performed well in the removal of heavy metals, especially Zn, *L. riparium* exhibited superior removal of Cu, indicating a greater affinity for certain ions. Moreover, *L. riparium* showed a marked decrease in TAN at 280 h, far exceeding the performance of *T. barbieri*. This suggests that while *T. barbieri* may initiate rapid contaminant removal during the initial stages of biofiltration, *L. riparium* excels in long-term removal processes. This suggests a time-dependent enhancement of TAN biofiltration in *L. riparium*, likely reflecting adaptive physiological responses possibly through cumulative uptake or microbial associations. The species-specific ability to uptake different metals was also proposed as a tool for bioremediation with mosses (Di Sansebastiano, 2020).

Biofiltration capacity was investigated in artificially established solutions mimicking hypothetical conditions of wastewater contaminated by human activity. We investigate HMs pollution using a moderate contamination of HMs possibly released by technological components, not lethal for most of plants, notably Cu, Pb and Zn. The presence of 4 μM CuSO_4_ is not an harmful contamination and we propose it is a standard background contamination. Nonetheless copper accumulation above such concentration can become toxic so it must be kept under control (Binesh and Venkatachalam, 2024). The proposed Pb and Zn contaminations represent moderate contamination issues and are within the lowest concentrations tested in plants toxicity studies (Seregin and Kozhevnikova, 2009; Meng et al., 2023).

Total Ammoniacal Nitrogen (TAN) starting concentration was determined considering an arbitrary contamination with 10% human urine.

These results underscore the importance of exposure duration and contaminant type when evaluating bryophyte performance under different biofiltration conditions. This is in line with previous evidence showing that bryophytes exhibit strong species-specificity in heavy metal uptake and can serve as efficient phytoremediators in water purification applications (Sabovljević and Sabovljević, 2020). This functional divergence supports the use of species consortia, combining fast-acting and long-term filter species to optimize BLSSs water purification modules.

Although aquatic bryophytes require a water medium, they can be integrated into compact, high-density cultivation modules to minimize total system volume, while exploiting their inherently high surface-to-volume ratio for efficient contaminant removal. Compared to higher aquatic plants, bryophytes offer a simpler morphology, no requirement for rooting substrate, and the ability to function effectively even in thin water layers or shallow-flow systems, reducing the volume of water needed for operation. Furthermore, their slow growth and low nutrient demand minimize the frequency of biomass turnover, lowering maintenance requirements and operational costs. This makes them suitable for modular bioreactor designs in which water volume is tightly controlled but contact surface with the biological filter is maximized. Furthermore, contaminated biomass from metal removal can be easily managed in BLSSs, as aquatic bryophytes form compact, non-motile mats that are simple to harvest and replace. Their modular cultivation allows safe containment and disposal or recovery of accumulated metals, reducing the risk of recontamination.

Taken together, these findings reveal complementary functional traits between *T. barbieri* and *L. riparium*. While the former is better suited for tasks involving photosynthetic productivity and resistance to light and temperature stress, the latter offers enhanced biofiltration capabilities for some heavy metals and especially for nitrogen compounds. The removal of nitrogenous waste, particularly ammonia and ammonium, is critical in BLSSs to prevent the accumulation of toxic compounds, ensure water quality, and maintain microbial and plant health in closed-loop environments (Drysdale et al., 2003). These differences highlight the potential of designing moss consortia that combine multiple species to optimize resource regeneration and detoxification in BLSSs.

Given their low maintenance requirements, high surface-to-volume ratio, and responsiveness to environmental conditions, aquatic bryophytes can serve as efficient biological components for air and water regeneration in space missions. Future research should focus on the impact of space-relevant stressors, such as ionizing radiation. In particular, long-term exposure studies on these species under combined light, thermal, and radiation stress will be essential to validate their performance and to assess cultivation scalability in operational BLSSs scenarios. Furthermore, integrating bryophytes into modular bioreactor designs could facilitate scalable and adaptive solutions for both extraterrestrial and terrestrial applications. These aspects remain among the major challenges in translating BLSSs prototypes to operational systems, as highlighted by recent integrative reviews on bioregenerative technologies for space exploration (Liu et al., 2021).

## 5 Conclusions and future perspectives

This study provides the first integrated evaluation of three aquatic bryophytes—*Taxiphyllum barbieri*, *Leptodictyum riparium*, and *Vesicularia montagnei*—for potential integration into Bioregenerative Life Support Systems (BLSSs). *T. barbieri* demonstrated the highest photosynthetic performance, pigment content, and antioxidant efficiency, making it well-suited for tasks requiring high physiological productivity and resistance to light and temperature variation. Conversely, *L. riparium* excelled in long-term removal of nitrogen compounds and specific heavy metals such as Cu, supporting its use as a specialized biofilter. These complementary functional traits suggest that mixed-species consortia could maximize both productivity and water purification efficiency in BLSS modules.

Future research should evaluate these species under prolonged exposure to space-relevant stressors, including ionizing radiation, microgravity, and fluctuating thermal and light regimes, to validate their long-term stability and scalability in operational settings. Additionally, the development of modular bioreactor systems incorporating bryophytes could enhance adaptability and resource regeneration in both extraterrestrial habitats and terrestrial water treatment applications.

## Data Availability

The original contributions presented in the study are included in the article/**Supplementary Material**, further inquiries can be directed to the corresponding author.
