# Supplementary material for: Aquatic bryophytes as biofilters and resource regenerators in Bioregenerative Life Support Systems: the moss on Mars project
Source: Front Plant Sci. 2025 Sep 23;16:1667463. doi: 10.3389/fpls.2025.1667463 (PMC12502980; doi:10.3389/fpls.2025.1667463)
Supplement: Supplementary file 1 [file Table1.docx]

Supplementary Material

# Supplementary Data

**Table S1.** Elements concentration measured as mg/L by ICP/AES in water before and after the supplement of contaminants. Mean value (MV) and standard deviation (SD) are shown.

|  |  | **Ca** | **Cd** | **Cu** | **Mn** | **Fe** | **Cr** | **Co** | **Ni** | **Pb** | **Al** | **K** | **Na** | **Mg** | **Ba** | **Sr** | **B** | **Be** | **Ga** | **Li** | **Se** | **Tl** | **Zn** |
| --- | --- | --- | --- | --- | --- | --- | --- | --- | --- | --- | --- | --- | --- | --- | --- | --- | --- | --- | --- | --- | --- | --- | --- |
| **Microfilter-sterilized water** | MV | 12.83 | 0.00 | 0.02 | 0.00 | 0.07 | 0.00 | 0.00 | 0.00 | 0.02 | 0.13 | 0.45 | 11.33 | 2.78 | 0.00 | 0.07 | 0.28 | 0.00 | 0.00 | 0.00 | 0.00 | 0.00 | 0.06 |
|  | SD | 0.23 | 0.00 | 0.01 | 0.00 | 0.00 | 0.00 | 0.00 | 0.00 | 0.01 | 0.01 | 0.00 | 0.42 | 0.05 | 0.00 | 0.01 | 0.05 | 0.00 | 0.00 | 0.00 | 0.00 | 0.00 | 0.02 |
| **Microfilter-sterilized water + HMs** | MV | 13.20 | 0.00 | 0.31 | 0.00 | 0.07 | 0.00 | 0.00 | 0.00 | 10.21 | 0.10 | 0.44 | 12.21 | 2.77 | 0.00 | 0.07 | 0.24 | 0.00 | 0.00 | 0.00 | 0.00 | 0.00 | 4.00 |
|  | SD | 0.09 | 0.00 | 0.00 | 0.00 | 0.01 | 0.00 | 0.00 | 0.00 | 2.05 | 0.00 | 0.01 | 0.20 | 0.04 | 0.00 | 0.01 | 0.01 | 0.00 | 0.00 | 0.00 | 0.00 | 0.00 | 0.12 |

**Table S2.** Results of two-way ANOVA testing the effects of species, environmental condition, and their interaction on Net-assimilation, transpiration, polyphenols, and DPPH scavenging activity.
Significance codes: Ns: non-significant, p ≤ 0.001 (***), p ≤ 0.01 (**), p ≤ 0.05 (*).

| **Parameter** | **Source** | **Sum Sq** | **Df** | **F value** | **P value** | **Significance** |
| --- | --- | --- | --- | --- | --- | --- |
| **Net-assimilation** | Species | 452.46 | 2 | 74.67 | 2.25×10⁻¹² | *** |
|  | Environment | 0.57 | 1 | 0.19 | 0.666 | ns |
|  | Specie × Environment | 17.69 | 2 | 2.92 | 0.069 | ns (trend) |
|  | Residuals | 90.90 | 30 |  |  |  |
| **Transpiration** | Species | 0.0957 | 2 | 48.58 | 3.91×10⁻¹⁰ | *** |
|  | Environment | 0.0028 | 1 | 2.83 | 0.103 | ns |
|  | Specie × Environment | 0.0018 | 2 | 0.93 | 0.405 | ns |
|  | Residuals | 0.0295 | 30 |  |  |  |
| **Chlorophyll a** | Species | 0.0386 | 2 | 202.09 | 5.75×10⁻¹⁰ | *** |
|  | Environment | 0.0125 | 1 | 131.35 | 8.05×10⁻^08^ | *** |
|  | Specie × Environment | 0.0254 | 2 | 133.03 | 6.45×10⁻^09^ | *** |
|  | Residuals | 0.001147 | 12 |  |  |  |
| **Chlorophyll b** | Species | 0.02218 | 2 | 678.8 | 4.522×10⁻^13^ | *** |
|  | Environment | 0.000067 | 1 | 4.11 | 0.065 | ns |
|  | Specie × Environment | 0.0032 | 2 | 100.01 | 3.2810⁻^8^ | *** |
|  | Residuals | 0.00019 | 12 |  |  |  |
| **Total chlorophylls** | Species | 0.164 | 2 | 452.28 | 5.036×10⁻¹^2^ | *** |
|  | Environment | 0.00094 | 1 | 5.208 | 0.041 | * |
|  | Specie × Environment | 0.024 | 2 | 66.83 | 3.12x10^-07^ | *** |
|  | Residuals | 0.0021 | 12 |  |  |  |
| **Carotenoids** | Species | 0.0132 | 2 | 259.7 | 1.32×10⁻¹⁰ | *** |
|  | Environment | 0.00005 | 1 | 2.26 | 0.1579 | ns |
|  | Specie × Environment | 0.0016 | 2 | 30.96 | 1.82x10^-05^ | *** |
|  | Residuals | 0.00030 | 12 |  |  |  |
|  | Species | 0.0927 | 2 | 7.66 | 0.003 | ** |
| **Polyphenols** | Environment | 0.1562 | 1 | 25.83 | 3.39×10⁻^5^ | *** |
|  | Specie × Environment | 0.1039 | 2 | 8.59 | 0.0015 | ** |
|  | Residuals | 0.1451 | 27 |  |  |  |
| **DPPH Scavening** | Species | 1.8272 | 2 | 2.3419 | 0.13846 | ns |
| **activity** | Environment | 1.0975 | 1 | 2.8134 | 0.11931 | ns |
|  | Specie × Environment | 2.5744 | 2 | 3.2996 | 0.07213 | ns |
|  | Residuals | 4.6813 |  |  |  |  |

**Table S3.** Results of three-way ANOVA testing the effects of species, environmental condition, time, and their interaction on Cu, Pb, Zn and TAN decrease %.
Significance codes: ns: non-significant, p ≤ 0.001 (***), p ≤ 0.01 (**), p ≤ 0.05 (*).

| **Parameter** | | | **Source** | | | **Sum Sq** | | | **Df** | | | **F value** | | | **P value** | | | **Significance** | | |  |
| --- | --- | --- | --- | --- | --- | --- | --- | --- | --- | --- | --- | --- | --- | --- | --- | --- | --- | --- | --- | --- | --- |
| **Cu decrease %** | | | Species | | | 666.0 | | | 1 | | | 85.33 | | | 2.26×10⁻^09^ | | | *** | | |  |
|  | | | Environment | | | 166.5 | | | 1 | | | 21.33 | | | 0.00011 | | | *** | | |  |
|  | | | Time | | | 7.5 | | | 2 | | | 0.48 | | | 0.624 | | | ns | | |  |
|  | | | Species × Environment | | | 41.6 | | | 1 | | | 5.33 | | | 0.0298 | | | * | | |  |
|  | | | Species x Time | | | 15.6 | | | 2 | | | 1.00 | | | 0.382 | | | ns | | |  |
|  | | | Environment x Time | | | 22.5 | | | 2 | | | 1.44 | | | 0.255 | | | ns | | |  |
|  | | | Species x Environment x Time | | | 5.2 | | | 2 | | | 0.33 | | | 0.719 | | | ns | | |  |
|  | | | Residuals | | | 187.3 | | | 24 | | |  | | |  | | |  | | |  |
| **Pb decrease %** | | | | Species | | | 0.04 | | | 1 | | | 0.03 | | | 0.86 | | | ns | | |
|  | | | | Environment | | | 7.34 | | | 1 | | | 5.60 | | | 0.03 | | | * | | |
|  | | | | Time | | | 21.44 | | | 2 | | | 8.19 | | | 0.002 | | | ** | | |
|  | | | | Species × Environment | | | 11.97 | | | 1 | | | 9.14 | | | 0.006 | | | ** | | |
|  | | | | Species x Time | | | 0.74 | | | 2 | | | 0.28 | | | 0.75 | | | ns | | |
|  | | | | Environment x Time | | | 6.27 | | | 2 | | | 2.39 | | | 0.11 | | | ns | | |
|  | | | | Species x Environment x Time | | | 0.45 | | | 2 | | | 0.17 | | | 0.84 | | | ns | | |
|  | | | | Residuals | | | 31.44 | | | 24 | | |  | | |  | | |  | | |
| **Zn decrease %** | | | | Species | | | 98.18 | | | 1 | | | 11.99 | | | 0.002 | | | ** | | |
|  | | | | Environment | | | 3.06 | | | 1 | | | 0.37 | | | 0.54 | | | ns | | |
|  | | | | Time | | | 0.18 | | | 2 | | | 0.01 | | | 0.98 | | | ns | | |
|  | | | | Species × Environment | | | 1.00 | | | 1 | | | 0.12 | | | 0.73 | | | ns | | |
|  | | | | Species x Time | | | 2.26 | | | 2 | | | 0.14 | | | 0.87 | | | ns | | |
|  | | | | Environment x Time | | | 60.44 | | | 2 | | | 3.69 | | | 0.04 | | | * | | |
|  | | | | Species x Environment x Time | | | 8.15 | | | 2 | | | 0.50 | | | 0.61 | | | ns | | |
|  | | | | Residuals | | | 196.42 | | | 24 | | |  | | |  | | |  | | |
| **TAN decrease %** | | | | Species | | | 54.9 | | | 1 | | | 3.06 | | | 0.08 | | | ns | | |
|  | | | | Environment | | | 57.6 | | | 1 | | | 3.21 | | | 0.07 | | | ns | | |
|  | | | | Time | | | 1515.7 | | | 2 | | | 42.19 | | | 1.88×10^⁻13^ | | | *** | | |
|  | | | | Species × Environment | | | 0.4 | | | 1 | | | 0.02 | | | 0.88 | | | ns | | |
|  | | | | Species x Time | | | 438.5 | | | 2 | | | 12.21 | | | 2.19×10^⁻05^ | | | *** | | |
|  | | | | Environment x Time | | | 174.8 | | | 2 | | | 4.86 | | | 0.009 | | | ** | | |
|  | | | | Species x Environment x Time | | | 67.7 | | | 2 | | | 1.88 | | | 0.16 | | | ns | | |
|  | | | | Residuals | | | 1526.8 | | | 85 | | |  | | |  | | |  | | |
